# Exaggerated increase of exercise-induced pulmonary artery pressure in systemic sclerosis patients predominantly results from left ventricular diastolic dysfunction

**DOI:** 10.1007/s00392-013-0594-x

**Published:** 2013-07-04

**Authors:** Michał Ciurzyński, Piotr Bienias, Katarzyna Irzyk, Maciej Kostrubiec, Zbigniew Bartoszewicz, Maria Siwicka, Marcin Kurzyna, Urszula Demkow, Piotr Pruszczyk

**Affiliations:** 1 Department of Internal Medicine and Cardiology, Medical University of Warsaw, Warsaw, Poland; 2 Department of Internal Medicine and Endocrinology, Medical University of Warsaw, Warsaw, Poland; 3 Department of Endocrinology, Warsaw Medical Research Centre of Polish Academy of Science, Warsaw, Poland; 4 Department of Dermatology, Medical University of Warsaw, Warsaw, Poland; 5 Department of Pulmonary Circulation and Thromboembolic Diseases, Medical Center of Postgraduate Education, European Health Centre, Otwock, Poland; 6 Department of Laboratory Diagnostics and Clinical Immunology of Developmental Age, Medical University of Warsaw, Warsaw, Poland

**Keywords:** Scleroderma, Echocardiography, Pulmonary hypertension, Diastolic dysfunction

## Abstract

**Objective:**

High prevalence of exaggerated pulmonary artery pressure response to exercise (EPAPR) was reported in patients with systemic sclerosis (SSc). However, pathophysiology of this phenomenon has not been well defined. Therefore, we evaluated the frequency and potential aetiology of EPAPR in SSc patients.

**Methods:**

We included 85 patients (79 female, 6 male, mean age 54.3 ± 13.9 years) with SSc. Transthoracic echocardiography followed by exercise Doppler echocardiography (EDE) were performed. A positive EDE was defined when at least 20 mmHg increase of tricuspid regurgitation peak gradient (TRPG) was recorded. Right heart catheterization (RHC) with exercise was performed in positive EDE patients and in subjects with resting TRPG >31 mmHg.

**Results:**

Resting TRPG >31 mmHg and/or positive EDE was found in 30 patients and they were referred to RHC. Finally, RHC was performed in 20 patients (16 pts resting TRPG >31 mmHg and 4 others normal resting TRPG and positive EDE). In 12 (60 %) of them an EPAPR with elevated pulmonary capillary wedge pressure (PCWP) was observed. Interestingly, mean left atrium (LA) diameter was greater in an EPAPR with elevated PCWP patients than in subjects with normal exercise response (39.36 ± 5.6 vs. 35.53 ± 3.48, *p* = 0.03). In EPAPR with elevated PCWP group greater mean value of E/E′ of mitral lateral annulus was observed (7.98 ± 3.35 vs. 6.27 ± 1.94, *p* = 0.03). In the univariate logistic regression analysis increased LA diameter was significant predictor of EPAPR with elevated PCWP (OR 1.199, 95 % CI 1.029–1.396, *p* = 0.019).

**Conclusions:**

Despite very well-known risk of PAH in systemic sclerosis patients, the excessive increase of PAP during exercise is more commonly caused by left ventricular diastolic dysfunction than pulmonary arterial vasculopathy.

## Introduction

Pulmonary hypertension (PH) is a well-known complication of systemic sclerosis and together with interstitial lung diseases is the leading cause of SSc-related deaths. The prevalence of pulmonary arterial hypertension in SSc patients is 7–12 % [1, 2]. Early and proper diagnosis of pulmonary hypertension (PH) in systemic sclerosis (SSc) patients is very important for their outcome, because patients with already advanced, symptomatic PH usually have poor prognosis [3–6]. In one randomized controlled trial was shown that therapeutic intervention at an early stage of PH may be beneficial [7]. Recent data suggest that there is an unexpectedly high prevalence of inappropriate pulmonary artery pressure (PAP) responses to exercise in patients with SSc [8–12]. However, its clinical significance is still under discussion. According to the latest European Guidelines definition of PH does not include exercise-induced PH mainly due to lack of sufficient data [3]. Some authors suggested that SSc patients with normal resting PAP and excessive exercise increase in PAP present an early stage of pulmonary arteriolar vasculopathy [13]. However, in this group of patients progressive cardiac fibrosis impairing left ventricular (LV) diastolic function was also reported [14, 15]. Therefore, we assumed that impaired LV diastolic function present in SSc patients is responsible for exaggerated PAP responses to exercise and can lead to post-capillary hypertension.

## Materials and methods

A group of 89 consecutive patients (82 females and 7 males, mean age 54.8 ± 13.6 years) with diagnosed SSc (mean disease duration 9.0 ± 12.4 years, range 1–45 years, median 6 years) were enrolled in this prospective study. The diagnosis of SSc was based on the American College of Rheumatology criteria [16]. Diffuse disease was found in 54 (61 %) patients and limited disease in the remaining 35 (39 %) patients. Clinical characterization of patients with SSc was performed according to the European Scleroderma Trial and Research (EUSTAR) recommendations [17].

We did not include patients with coronary artery disease (angina pectoris, previous myocardial infarction, ECG and/or echocardiographic signs of myocardial ischemia), severe systemic hypertension, LV hypertrophy (intraventricular septum or posterior wall >11 mm at echocardiographic examination) and with significant valvular heart disease. Restrictive LV filling at resting echocardiography with an average E/E′ >13 (or septal E/E′ ≥15 or lateral E/E′ >12) was also an exclusion criterion [18]. We excluded patients with significant pulmonary dysfunction defined as forced vital capacity (FVC) and total lung capacity values <60 % predicted and/or the forced expiratory volume in one second to vital capacity ratio (FEV1/VC) <70 % predicted. We also did not include patients with significant fibrotic changes in the lung by high-resolution computed tomography (Kazerooni fibrosis score ≥16). An impaired renal function with creatinine clearance calculated by Modification of Diet in Renal Disease Study Group (MDRD) formula below 60 ml/min was also an exclusion criterion.

As a control group we assessed 21 age and sex-matched subjects (18 females, 3 males, mean age 49.3 ± 10.5 years) with similar profile of coexisting systemic hypertension and its treatment as the study group. These subjects had no data for pulmonary diseases and presented no echocardiographic evidence of structural heart disease.

All patients and control subjects gave informed consent, and the protocol of the study was approved by the local Institutional Ethics Committee (No. KB66/2006).

## Echocardiography

Echocardiographic examination was performed with Philips iE 33 (Andover, Md., USA) with 2.5–3.5 MHz transducers. Using continuous-wave Doppler echocardiography, the tricuspid regurgitation peak gradient (TRPG) was calculated according to the simplified Bernoulli’s equation. According to European Society of Cardiology (ESC) criteria PH was suspected when TRPG >31 mmHg (tricuspid regurgitant velocity >2.8 m/s) [5]. In order to assess the right ventricular function using the one-dimensional M-mode echocardiography, the tricuspid annular peak systolic excursion (TAPSE) was measured. The LV ejection fraction (EF) was calculated according to the modified Simpson rule using apical four- and two-chamber views [19].

### Assessment of left ventricular diastolic function

Mitral valve inflow (MVF) was recorded in the apical four-chamber view with Doppler gate positioned in the LV on the level of the mitral valve edges. The following parameters were evaluated: peak velocity of the early inflow phase (E), peak velocity of the atrial inflow phase (A) and E/A ratio.

Tissue Doppler imaging (DTI) was performed in the apical views to acquire mitral annular velocities. Lateral annulus early diastolic velocity (Mit E′ lateral) and septal annulus early diastolic velocity (Mit E′ septal) were measured. Moreover, mitral E/E′ lateral and septal were calculated. E/E′ >13 (or septal E/E′ ≥15 or lateral E/E′ >12) was defined as abnormal [18].

### The exercise echocardiography protocol and the methodology for measuring right ventricular systolic pressure at rest and following exercise

The patient then performed a standard exercise on a treadmill according to the Bruce protocol until 85 % of the maximum heart rate was achieved. The exercise was then stopped and the patient resumed the left lateral position as soon as possible. In the apical four-chamber view, using continuous-wave Doppler, the peak velocity of the tricuspid regurgitant wave was recorded and the TRPG was calculated. The TRPG was recorded within 1 min after stopping the exercise. PH was suspected when TRPG at rest exceeded 31 mmHg (Vmax >2.8 m/s) or increased by at least 20 mmHg versus baseline following exercise [13]. Patients with suspicion of PH were referred to right heart catheter (RHC).

### Hemodynamics assessment of the right heart

Cardiac catheterization was performed within 6 weeks of exercise echocardiography according to the previously described protocol [20]. The Swan-Ganz catheter was passed under fluoroscopic guidance to the pulmonary artery. The following variables were recorded: PAP (systolic, diastolic and mean), mean pulmonary capillary wedge pressure (PCWP) and mean right atrial pressure.

Cardiac output was determined by thermodilution injecting cooled saline until a variability of <10 % was achieved in three consecutive measurements. The vascular resistance values in the pulmonary and systemic circulation were calculated using typical formulas. The exercise test was performed on a cyclo ergometer in the lying position, aiming to achieve the maximum load of 125 W. After 5 min of maximal tolerated exercise the same measurements as those at rest were performed. A special attention has been paid to the reliable measurement of PCWP. Tip of the catheter was placed in distal part of the pulmonary arterial tree and balloon was carefully filled with increasing volume of air to avoid over wedge phenomenon. The measurement was accepted, when rapid drop in PAP has been obtained and biphasic pressure curve with respiratory variation typical for wedge pressure was recorded. Digitized mean values of PCPW were used for calculations at rest and at exercise. We found it impossible to measure PCPW at exercise using breath-hold technique or extract end-expiratory values, because of high respiratory rate and important fluctuations in intrathoracic pressure.

In accordance with the ESC recommendations, PAH at rest was diagnosed when mPAP was ≥25 mmHg and PCWP did not exceed 15 mmHg [5]. Exaggerated pulmonary artery pressure response to exercise (EPAPR) with normal PCWP was identified when mPAP during exercise exceeded 30 mmHg in the presence of normal PCWP. The venous PH at rest was diagnosed when mPAP at rest was ≥25 mmHg and PCWP exceeded 15 mmHg [5]. Exaggerated pulmonary artery pressure response to exercise (EPAPR) with elevated PCWP was diagnosed when mPAP during exercise exceeded 30 mmHg and PCWP exceeded 20 mmHg [21].

### Blood sampling and assays

Fasting blood samples were collected by venipuncture, centrifuged and sera were stored at −70 °C until assayed. The concentration of NT-proBNP was analyzed on Elecsys 2010 automatic analyzer (Roche Diagnostics, Basel, Switzerland). Serum NT-proBNP concentration higher than 125 pg/ml was regarded as abnormal as indicated by the producer.

### Statistical analysis

Data characterized by a normal distribution are expressed as a mean followed by a standard deviation. Biochemical parameters are expressed both as a mean followed by a standard deviation and as a median with a range. Patients with SSc and controls were compared with Wilcoxon test depending on the character of parameters distribution. For categorical variables, the differences between the groups were compared with χ ^2^ test or Fisher’s exact test. Correlations between echocardiographic and biochemical variables were evaluated by Spearman’s correlation coefficients. Logistic regression analysis was done to assess the odds ratio for abnormal exercise increase in post-capillary PAP.

An analysis was performed using a statistical software package (SAS 9.2). *p* < 0.05 was considered statistically significant.

## Results

None of the patients enrolled in our study had any clinical symptoms at rest suggestive for PH. Fifteen (17 %) subjects complained mild or moderate exertional dyspnoea. Due to orthopaedic conditions preventing from undergoing an adequate physical exercise 4 (4.5 %) patients were excluded. Finally, a total of 85 SSc patients performed exercise test.

The general characteristics of 85 SSc patients and control group undergoing exercise echocardiography are summarized in Table 1.

**Table 1:** The general parameters in the SSc and the control groups

Parameter	SSc patients (*n* = 85)	Control subjects (*n* = 21)	*p* value
Age (years)	54.3 ± 13.9	49.3 ± 10.5	0.09
Gender (F/M), no.	79/6	18/3	0.38
Body surface area (m^2^)	1.73 ± 0.26	1.72 ± 0.18	0.7
Heart rate (bpm)	73.47 ± 9.45	76.21 ± 12.38	0.3
Blood pressure systolic (mmHg)	125.7 ± 18.8	123.3 ± 15.4	0.57
Blood pressure diastolic (mmHg)	76.4 ± 11	82.3 ± 12	0.02
Systemic hypertension (%)	25 (29 %)	6 (29 %)	0.2

### Treatment

Angiotensin-converting enzyme inhibitors (ACE-I) received 23 (27 %) SSc patients, angiotensin II receptor antagonists-5 (6 %), beta blockers-7 (8 %), diuretics-12 (14 %), calcium channel blockers-20 (23 %). Due to the progression of SSc 12 (14 %) patients received immunosuppressant agents (glucocorticoids and cyclophosphamide). We did not find significant differences in the use of the cardiovascular drugs between SSc and control group.

The main clinical, pulmonary function and serological findings of SSc patients are shown in Table 2.

**Table 2:** Clinical, pulmonary function and serological data of 85 SSc patients

Characteristics	Mean	Median, range
Disease duration (years)	9.0 ± 12.4	5.0 (1–25)
Rodnan score	6.4 ± 6.7	4.0 (1–35)
FVC, % predict	100.4 ± 18.3	
FEV_1_, % predict	94.7 ± 19.7	
FEV_1_/FVC, % predict	79.8 ± 8.2	
TLC, % predict	101.9 ± 17.5	
DLCO, % predict (*n* = 59)	70.7 ± 19.1	
Autoantibodies	No	%
ANA positive	79	93
ACA positive	28	33
Anti- Topo I	40	48

*FVC* forced vital capacity, *FEV1* forced expiratory volume in 1 s, *TLC* total lung capacity, *DLCO* carbon monoxide diffusing capacity

### Echocardiographic data

In Table 3 the echocardiographic parameters in SSc group and controls are presented.

**Table 3:** Echocardiographic parameters in SSc patients and controls

Variables	SSc patients (*n* = 85)	Controls (*n* = 21)	*p* value
EF (%)	65 ± 5.1	67 ± 2.52	0.01
LA	32.3 ± 4.52	31.1 ± 3.46	0.53
RV/LV 4 chambers	0.73 ± 0.12	0.66 ± 0.08	0.01
TAPSE (mm)	22.2 ± 3.23	24.14 ± 2.37	0.01
MAPSE (mm)	15.4 ± 2.4	16.3 ± 1.8	0.05
Mitral E/A	0.96 ± 0.3	1.2 ± 0.3	0.002
Mit E/E′ lateral mitral annulus	7.55 ± 2.85	6.87 ± 2.3	0.4

*EF* ejection fraction, *LA* left atrium, *RV* right ventricle, *LV* left ventricle, *TAPSE* tricuspid annulus plane systolic excursion, *MAPSE* mitral annulus plane systolic excursion

The SSc patients presented lower mean value of mitral E/A. We did not find significant differences between the mean value of LA diameter and E/E′.

The main parameters recorded before and after exercise test in SSc patients and controls are shown in Table 4.

**Table 4:** The main parameters before and after exercise test in SSc and controls

Parameter	SSc (n = 85)	Controls (n = 21)	P value
HR (1/min)	84.4 ± 17.3	85.3 ± 17.2	0.68
Max exercise HR (1/min)	152.8 ± 22.9	165.3 ± 11.7	0.01
Workload (METS)	8.5 ± 2.66	10.91 ± 2.89	0.0008
HR max (%)	93.3 ± 13.8	96.6 ± 5.3	0.5
Resting TRPG (mmHg)	26.9 ± 6.3	17.8 ± 4.1	<0.0001
Exercise TRPG (mmHg)	39.0 ± 11.1	22.4 ± 8.4	<0.0001
Δ TRPG (mmHg)	12.5 ± 8.1	7.6 ± 4.5	0.02

*TRPG* tricuspid regurgitant peak gradient

The mean resting and exercise TRPG values and Δ TRPG were significantly higher in SSc patients than in controls.

Significant correlations between exercise TRPG and echocardiographic indices are listed in Table 5.

**Table 5:** Significant correlations between exercise TRPG and echocardiographic parameters

Parameter	*r*	*p*
LA	0.4	0.001
MAPSE	−0.33	0.008
E′ Lateral mitral annulus (cm/sek)	−0.31	0.01
Mit E/E′ Lateral mitral annulus	0.3	0.01

Moreover the mean value of Δ TRPG correlates with LA diameter (*r* = 0.33, *p* = 0.008)

### Exercise echocardiography

**Fig. 1:**
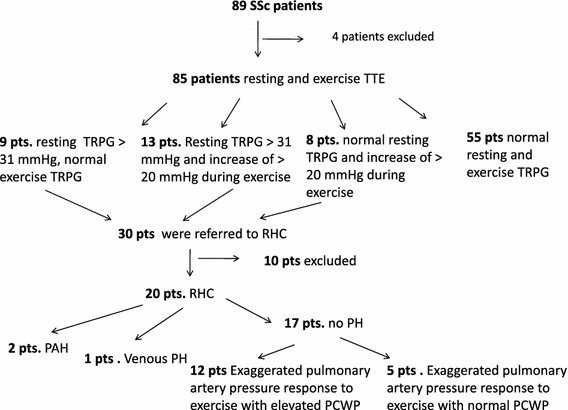
Qualification of patients for right heart catheterization; *TTE* transthoracic echocardiography, *TRPG* tricuspid regurgitation peak gradient, *RHC* right heart catheterization, *PAH* pulmonary arterial hypertension, *PH* pulmonary hypertension, *PAP* pulmonary artery pressure

Thirty (35 %) patients with TRPG exceeding 31 mmHg at rest and/or an exercise increase in TRPG >20 mmHg were referred for RHC. Eight refused to undergo the procedure, one patient could not performed the procedure due to a worsening of general condition and one patient died due to bleeding from oesophageal varices while awaiting the procedure. Finally, RHC was performed in 20 patients (16 pts resting TRPG >31 mmHg and four with normal resting TRPG and EPAPR). In 12 (60 %) of them an EPAPR with elevated PCWP was observed (mean exercise mPAP 41.8 ± 13.2 mmHg, mean exercise PCWP 25.1 ± 4.8 mmHg). In remaining two PAH, one pulmonary venous hypertension and five pts an EPAPR with normal PCWP was diagnosed (Fig. 1). In all four patients with normal resting TRPG and EPAPR, elevated PCWP during exercise was observed.

Parameters obtained during RHC in SSc patients are listed in Table 6.

**Table 6:** RHC parameters in SSc patients

Parameter (*n* = 20)	Mean	±SD	Median	Range
s PAP (mmHg)	33.2	6.6	32.5	23.0–48.0
m PAP (mmHg)	21.1	4.2	20.5	15.0–29.0
s PAP exercise (mmHg)	59.7	18.8	55.0	38.0–115.0
m PAP exercise (mmHg)	43.0	12.7	41.0	30.0–82.0
PVR (Wood U)	2.02	0.9	1.79	1.07–4.46
PCWP (mmHg)	11.2	3.4	10.5	7.0–20.0
PCWP exercise (mmHg)	22.6	6.1	23.0	17.0–32.0

*sPAP* systolic pulmonary artery pressure, *mPAP* mean pulmonary artery pressure, *PVR* pulmonary vascular resistance, *PCWP* pulmonary capillary wedge pressure

In Table 7 clinical, echocardiographic and biochemical parameters in SSc patients with EPAPR with elevated PCWP and in the group of normal resting end exercise TRPG are listed.

**Table 7:** Clinical, echocardiographic and biochemical parameters in patients with EPAPR with elevated PCWP and in normal resting end exercise TRPG

Parameter	EPAPR with elevated PCWP (*n* = 12)	Normal TRPG at rest end during exercise (*n* = 55)	*p* value
Age	59.6 ± 7.9	51.6 ± 13.3	0.03
LA (mm)	39.36 ± 5.6	35.53 ± 3.48	0.03
TAPSE (mm)	19.86 ± 3.02	22.26 ± 2.75	0.01
MAPSE (mm)	14.6 ± 1.63	15.62 ± 1.98	0.05
E/E′ mitral annulus	7.98 ± 3.35	6.27 ± 1.94	0.03
NT-proBNP (pg/ml)*	Median, range		0.01
	130.8, 57–2195	80.8, 14.07–584.4	

*TRPG* tricuspid regurgitant peak gradient

* Wilcoxon test

The mean LA diameter was significantly increased in SSc patients with EPAPR with elevated PCWP than in subjects with normal PAP values. Also the mean value of E/E′ was higher in the former.

In the univariate logistic regression analysis we identified parameters that predicted EPAPR with elevated PCWP (Table 8).

**Table 8:** Parameters that increase the chance of EPAPR with elevated PCWP

Parameter	OR	95 % CI	P value
TAPSE, 1 mm decrease	1.386	1.074–1.788	0.012
LA diameter, 1 mm increase	1.199	1.029–1.396	0.019
Age, 1 year increase	1.06	1.002–1.121	0.04

TAPSE, LA diameter, and patients’ age are the parameters that increase the chance of EPAPR with elevated PCWP

## Discussion

Some data underline that an excessive increase in PAP during exercise cannot be regarded as the norm [22, 23]. It was even postulated that this is an early preclinical stage of PH. Moreover, there are also reports showing beneficial effects of bosentan treatment in asymptomatic patients, but with excessive increase in PAP during exercise [24]. Steen and colleagues [13] evaluated 54 patients with SSc who underwent exercise echocardiography. They showed increase in exercise systolic PAP greater than 20 mmHg in 44 % of them. Also, Alkotob et al. [25] found an increase in exercise systolic PAP in 46 % of the 65 patients with SSc. Moreover, in a paper published by Pignone et al. [26] authors showed exertional increase in systolic PAP above 40 mmHg in 18 (67 %) of 27 patients with SSc. In a recently published paper Gargani et al. [12] exercise Doppler echocardiography revealed significant exercise-induced increase in PAP in 69 (42 %) among 164 SSc patients with normal resting PAP. Exercise Doppler echocardiography is useful not only in patients with SSc but also in another population. Ha et al. [27] examined during exercise echocardiography 396 patients with normal left ventricular systolic function. They revealed that 135 (35 %) of them had systolic PAP >50 mmHg and it was associated with E/E′ ratio.

Using standard rest and exercise echocardiography we identified 30 patients with possible PH. Finally, RHC was performed in 20 patients. Of these, four (20 %) patients were qualified to the hemodynamic study because of the excessive increase in PAP during exercise, with normal resting values of TRPG. During the RHC 12 (60 %) patients showed an EPAPR with elevated PCWP, while only in two PAH was eventually diagnosed. Based on these observations, it seems that Doppler echocardiography is a useful method to identify abnormal exercise-increased PAP in patients with SSc. However, to determine the type of PH requires cardiac catheterization. Only limited evidence indicates that LV diastolic dysfunction may be the key mechanism responsible for the inappropriate exercise-increased PAP. Kovacs and colleagues [9] performed echocardiographic exercise test in 52 patients with connective tissue disease (26 patients with SSc). In 26 patients, despite normal values of systolic PAP at rest, exercise systolic PAP was increased up to >40 mmHg. Twenty-one patients underwent RHC, which confirmed the normal systolic PAP at rest and exercise systolic PAP increased to >40 mmHg in 19 of them. Of these eight cases during RHC had elevated PCWP >20 mmHg. Saggar et al. [28] reported a group of 57 SSc patients who had normal resting hemodynamics and underwent subsequent exercise RHC. An inappropriate exercise-induced venous increase in PAP was reported in 12 (21 %) of them. D’Alto et al. [8] evaluated 172 patients with SSc without PH and without significant LV diastolic dysfunction (E/E’ <15). In the SSc group authors found significantly higher mean value of resting systolic PAP and systolic PAP recorded after exercise. Interestingly, in patients with impaired diastolic function (the lowest quartile of the values of E′/A’) the average value of systolic PAP recorded after exercise was significantly higher than in subjects with preserved diastolic function (the highest quartile of the values of E′/A’) (*p* = 0.015). In recently published paper, Hager et al. [29] performed RHC in 173 SSc patients when resting echocardiographic PAP were <40 but >40 mmHg after exercise. LV diastolic dysfunction was diagnosed in 47 patients. Our findings are consistent with observations by D’Alto and Hager et al. Moreover, we observed significant correlations between exercise TRPG and echocardiographic indices of LV diastolic dysfunction in SSc patients. The mean value of exercise TRPG significantly positively correlated with the LA dimension, the E/E′ lateral mitral annulus ratio and negatively with a mean E’ lateral mitral annulus velocity. We have also demonstrated a positive correlation between the change of TRPG and LA dimension. These correlations indicate a potential link between rest and exercise TRPG and an impaired LV relaxation. We tried to define parameters potentially useful for differentiating patients with EPAPR with elevated PCWP. Thus, this group was characterized by significantly higher mean LA dimension and greater value of the E/E’ lateral mitral annulus ratio. These observations may support the hypothesis that diastolic dysfunction is one of the major causes of EPAPR with elevated PCWP. It seems that progressive diastolic dysfunction does not impair PH at rest; however, it can contribute to an abnormal EPAPR with elevated PCWP. Moreover, there are evidences that serum NT-proBNP level may be useful for detection of LV diastolic dysfunction [30]. In our SSc patients with EPAPR with elevated PCWP we have found significantly higher concentrations of serum NT-proBNP level, suggesting neurohormonal activation. In univariate, logistic regression analysis we identified factors that increase the chance of EPAPR with elevated PCWP, such as LA diameter (1.199, 95 % CI 1.029–1.396, *p* = 0.019), confirming the importance of diastolic dysfunction. Our study is one of the first to show that among SSc patients exaggerated pulmonary artery pressure responses to exercise is predominantly caused by impaired LV relaxation.

### Study limitations

Eight SSc patients had not consented to RHC which may have influenced the results. Moreover, RHC was performed within 6 weeks after exercise echocardiography which might have resulted in changes of hemodynamic status of patients during awaiting the procedure. On the other hand, the clinical condition of patients while awaiting the RHC was stable and drug treatment was not modified.

The control group was 5 years younger than the SSc group (54.3 ± 13.9 vs. 49.3 ± 10.5 years). Although the difference was not significant *p* = 0.09.

## Conclusions

Despite very well-known risk of PAH in systemic sclerosis patients, the excessive increase of PAP during exercise is more commonly caused by left ventricular diastolic dysfunction than pulmonary arterial vasculopathy.

## Abbreviations list

ACE-I: Angiotensin-converting enzyme inhibitors
DTI: Tissue Doppler imaging
EDE: Exercise Doppler echocardiography
EPAPR: Exaggerated pulmonary artery pressure responses to exercise
ESC: European society of cardiology
EUSTAR: European Scleroderma Trial and Research
FEV1/VC: Forced expiratory volume in one second to vital capacity ratio
FVC: Forced vital capacity
LA: Left atrium
LV: Left ventricle
MAPSE: Mitral annular peak systolic excursion
MDRD: Modification of diet in renal disease study group
MVF: Mitral valve inflow
NT-proBNP- N: Terminal pro-brain natriuretic peptide
PAP: Pulmonary arterial pressure
PCWP: Pulmonary capillary wedge pressure
PH: Pulmonary hypertension
RHC: Right heart catheterization
SSc: Systemic sclerosis
TAPSE: Tricuspid annular peak systolic excursion
TRPG: Tricuspid regurgitation peak gradient
